# Production Variability and Categorical Perception of Vowels Are Strongly Linked

**DOI:** 10.3389/fnhum.2019.00096

**Published:** 2019-03-22

**Authors:** Sara-Ching Chao, Damaris Ochoa, Ayoub Daliri

**Affiliations:** Speech and Hearing Science, College of Health Solutions, Arizona State University, Tempe, AZ, United States

**Keywords:** speech, perception, variability, speech motor control, vowels

## Abstract

Theoretical models of speech production suggest that the speech motor system (SMS) uses auditory goals to determine errors in its auditory output during vowel production. This type of error calculation indicates that within-speaker production variability of a given vowel is related to the size of the vowel’s auditory goal. However, emerging evidence suggests that the SMS may also take into account perceptual knowledge of vowel categories (in addition to auditory goals) to estimate errors in auditory feedback. In this study, we examined how this mechanism influences within-speaker variability in vowel production. We conducted a study (*n* = 40 adults), consisting of a vowel categorization task and a vowel production task. The vowel categorization task was designed—based on participant-specific vowels—to estimate the categorical perceptual boundary (CPB) between two front vowels (/ε/ and /æ/). Using the vowel production data of each participant, we calculated a variability-based boundary (VBB) located at the “center of mass” of the two vowels. The inverse of the standard deviation of a vowel distribution was used as the “mass” of the vowel. We found that: (a) categorical boundary was located farther from more variable vowels; and (b) the calculated VBB (i.e., the center of mass of the vowels) significantly and positively correlated with the estimated categorical boundary (*r* = 0.912 for formants calculated in hertz; *r* = 0.854 for formants calculated in bark). Overall, our findings support a view that vowel production and vowel perception are strongly and bidirectionally linked.

## Introduction

A large body of literature indicates that the speech production and speech perception systems interact in many intricate ways (Galantucci et al., 2006; Guenther, 2006; Tatham and Morton, 2006; Hickok, 2012; Perkell, 2012). Recent functional imaging studies have reported that motor regions—classically believed to be involved in movement production—are active during speech perception tasks (Wilson et al., 2004; Skipper et al., 2005; D’Ausilio et al., 2009; Grabski et al., 2013; Schuerman et al., 2017). Similarly, auditory regions—classically believed to be involved in speech perception—are active during speech production (Tourville et al., 2008; Hickok, 2012; Niziolek et al., 2013; Skipper et al., 2017). In fact, in a series of studies, we provided behavioral and electrophysiological evidence that the auditory system is prepared for its roles in speech monitoring several 100 ms prior to speech initiation (Daliri and Max, 2015, 2016, 2018; Merrikhi et al., 2018). Overall, the dynamic relationship between auditory and motor regions plays an important role in both speech production and speech perception (Houde and Nagarajan, 2011; Guenther and Vladusich, 2012; Hickok, 2012; Houde and Chang, 2015; Daliri et al., 2018).

Current theoretical models of speech production suggest that vowel production is strongly reliant on internally represented speech goals (Houde and Nagarajan, 2011; Hickok, 2012; Guenther, 2016). Although the exact nature of the speech goals is unclear, it has been suggested that the speech motor system (SMS) may use perceptual goals (e.g., auditory goals) to determine errors in its motor output (Perkell et al., 1997, 2008; Perkell, 2012; Guenther, 2016). These models posit that during production, the SMS compares auditory feedback of the produced speech with its auditory goals; when the auditory feedback resides outside the auditory goals (i.e., auditory error), the SMS generates corrective motor responses to reduce the perceived error. One prediction of such conceptualization is that speakers with smaller auditory goals would have smaller production variability. Because auditory goals cannot be measured directly, auditory acuity measures—estimated *via* speech discrimination tasks—have been adopted as proxies for auditory goals (Perkell et al., 2004a; Villacorta et al., 2007; Feng et al., 2011; Perkell, 2012; Daliri et al., 2013; Franken et al., 2017). In a speech discrimination task, speakers are asked to discriminate between speech sounds with subtle acoustic differences; therefore, discrimination tasks measure speakers’ ability to distinguish small changes in auditory input (i.e., auditory acuity). In support of this prediction, a few studies have examined the relationship between auditory acuity and vowel production (Perkell et al., 2004a, 2008; Franken et al., 2017). These studies have shown that speakers with better auditory acuity—typically interpreted as smaller auditory goals regions—produce more consistent vowels (i.e., smaller within-vowel variability).

This type of interpretation is in line with phonetic theories that rely on local constraints (e.g., in articulatory-acoustic-perceptual space) to explain how phonological systems emerge (Stevens, 1989; Stevens and Keyser, 2010). However, it has been argued that phonological systems can also emerge based on global constraints (e.g., maximizing distance between different phonemes; Liljencrants et al., 1972) or a combination of local and global constraints (Schwartz et al., 1997, 2005). It is conceivable, therefore, to argue that the SMS may also use global constraints in addition to local constraints to more accurately produce vowels or phonemes, in general. In fact, emerging evidence suggests that the SMS may rely on perceptual knowledge of vowel categories to estimate errors in auditory feedback (Niziolek and Guenther, 2013; Bourguignon et al., 2014, 2016; Lametti et al., 2014a). For example, in a seminal study, Niziolek and Guenther (2013) showed that real-time auditory feedback perturbations (shifts in formant frequencies) of productions that were closer to the edge of the vowel category elicited larger compensatory responses relative to identical perturbations of productions closer to the center of the vowel (far from the edge of the vowel boundary). These results suggested that the SMS may use the perceptual boundary between two adjacent vowels—in addition to auditory goals—to determine errors in its output. Certainly, auditory feedback perturbations provide valuable insights into the mechanisms of error calculation in response to altered auditory feedback; however, it is not clear how this type of error calculation influences within-speaker variability in vowel production with normal, unaltered auditory feedback.

Generally, perceptual distinctiveness of two phonemes depends on the distance of each of the phonemes from their joint categorical boundary. For example, two cross-boundary tokens (e.g., /ε/ and /æ/) that are close to the categorical boundary between them are less distinct than two tokens that are far from the categorical boundary and close to their centroids (Kuhl, 1991; Kuhl et al., 2007; Goldstone and Hendrickson, 2010). In this study, we examined whether the categorical boundary between two adjacent vowels is related to variability of the vowels—in two adjacent vowels, the vowel closer to the categorical boundary is less variable than the vowel farther from the categorical boundary. In other words, each vowel pushes the perceptual boundary away based on the inverse of its variability. In an analogy to physics, two adjacent vowels can be considered two connected masses, and the “mass” of each vowel can be determined by the inverse of the variability of the vowel distribution. Based on this analogy, we hypothesized that variabilities of two adjacent vowels may co-vary with the categorical boundary between the vowels, and the “center of mass” of the vowel categories correlates with the categorical perceptual boundary (CPB). To test this hypothesis, we conducted a standard categorical perception task to estimate the perceptual boundary between /ε/ and /æ/. Given that our goal was to examine each participant’s perception in relation to the participant’s production variability, we used a participant-specific speech sample to generate a participant-specific vowel continuum for the categorical perception task. We also conducted a vowel production task (/ε/ and /æ/) and calculated the variability of each of the vowels and combined the variabilities to construct a theoretical variability-based boundary (VBB; the center of mass of vowel distributions). We found that the calculated VBB positively and strongly correlated with the perceptual boundary.

## Materials and Methods

### Participants

Forty healthy adult speakers (29 female participants; *M*_age_ = 24.07 years, *SD*_age_ = 4.67 years; age range 18.42–43.01 years) participated in this study. Participants were native speakers of American English with no history of neurological, psychological, speech-language disorders, and hearing disorders (pure tone hearing threshold ≤20 dB HL at octave frequencies from 250 to 8,000 Hz). The Institutional Review Board at Arizona State University approved all study protocols. Participants signed a consent form prior to participation in the experiment. Participants were recruited from a participant pool of undergraduate students.

### Procedure

Participants were seated inside a sound booth in front of a computer monitor. A microphone (SM58, Shure) mounted on a stand was placed 15 cm from the corner of the participant’s mouth (at ~45° angle). The microphone signal was amplified (Tubeopto 8, ART) and digitized (at 48,000 Hz sampling rate) *via* an audio interface (Ultralite Mk3 hybrid, MOTU). Output signals of the audio interface were then amplified (Pro Rx1602, Eurorack) and played back to the participant *via* insert earphones (ER-1, Etymotic Research Inc.). The input-output level was calibrated prior to each experiment to ensure that the intensity of the played-back signal was 5 dB greater than the microphone signal.

Each participant completed the study in one session that took less than 30 min. Participants completed a practice task in which they overtly produced monosyllabic consonant-vowel-consonant (CVC) words (e.g., “head”). The practice task (30 trials) was used to familiarize participants with the setup and to train them to pronounce target words within a desired intensity (70–80 dB SPL) and duration (400–600 ms; based on the voiced segments) range. After each trial, participants received visual feedback regarding their intensity and duration. Next, participants completed a *vowel production task* that was similar to the practice task. Participants produced CVC words that contained /ε/ or /æ/ (30 trials of each vowel). The order of words (vowels) was randomized. In this task, if participants produced words within the desired intensity and duration ranges, they did not receive visual feedback.

**Figure 1:**
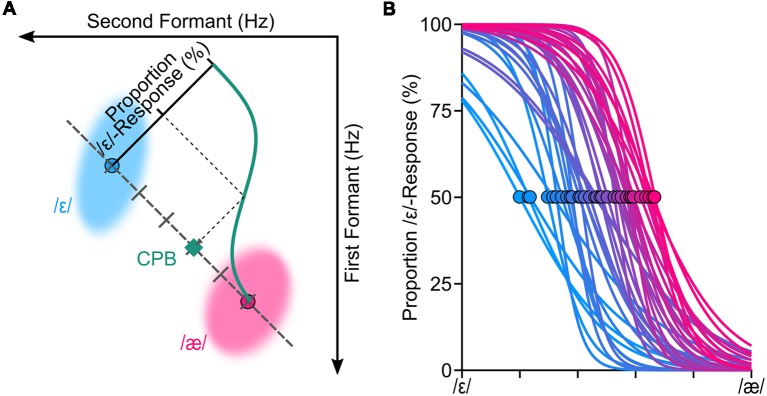
We conducted a standard categorical perceptual task to estimate the categorical perceptual boundary (CPB) between /ε/ and /æ/ for each participant. Panel **(A)** shows the procedure for estimating the CPB (at 50%) through fitting a logistic psychometric function to the perceptual data for one representative participant. To generate speech stimuli for the categorical perception task, we manipulated participants’ own production samples (formant shifted) along the line connecting participant-specific centroids of /ε/ and /æ/. The shaded areas (ellipses) in **(A)** correspond to the spread of the formants in hertz (two standard deviations from the mean of principle components) of /ε/ and /æ/ for this representative participant. Psychometric functions were successfully fitted (*R*^2^ > 0.85) to all participants’ responses **(B)**. The circles in **(B)** correspond to where CPBs are located for all participants.

Upon completion of the vowel production task, for each produced word in the vowel production task, we extracted the first formant frequency (F1) and the second formant frequency (F2) from vowels of each word. We used Audapter—a publicly available software for formant tracking and manipulation—to automatically extract the formant frequencies (Cai, 2015). Audapter is a MATLAB-based software package that its source code is implemented in C++ and consists of several speech processing blocks, including formant tracking and formant manipulation. Audapter uses linear predictive coding (LPC) analysis and dynamic programming to track formant frequencies. We used LPC order of 17 for male participants and 15 for female participants. The speech data was recorded at 48,000 Hz and down-sampled to 16,000 Hz to reduce computational loads. To improve formant-tracking accuracy, we supplied Audapter with participant-specific initial values for F1 and F2 (in Hz) that were estimated based on the practice trials. Audapter uses smoothed short-term energy criteria in combination with heuristic rules to determine onset and offset of voiced segments and to initiate formant tracking and formant manipulations. After the vowel production task, a custom written algorithm used onset and offset values determined by Audapter and extracted the average formant values (in Hz) in a window placed on the center of the segment (10%–90% into the length of the segment). Using F1-F2 coordinates, the algorithm used the Euclidian distance to determine the token closest to the median of the vowel /ε/ and the median of the vowel /æ/ (hereafter called median productions). In other words, *median productions* of a given participant were words produced by the participant that were closest to the center of the distribution of the vowel /ε/ and the center of distribution of the vowel /æ/ of the participant (in F1-F2 coordinates). Using these participant-specific median productions, we generated a set of six or seven equally spaced stimuli (formant shifted CVC words) along the line connecting the median /ε/ and the median /æ/ for each participant. Given that samples were generated based on participant specific speech, the duration of stimuli were different for different participants. The duration of the voiced segment of stimuli ranged from ~382 ms to ~627 ms (*M* = 472 ms, *SD* = 39 ms). However, for a given participant, only the vowel portions of the stimuli (words) were different, as the stimuli were generated based on the participant-specific median production by shifting F1 and F2 of the median /ε/ (using offline formant shift of Audapter). The stimuli were designed such that the vowel of the first stimulus coincided with the median /ε/ and the vowel of the last stimulus coincided with the median /æ/. Figure 1A shows a set of six stimuli for a representative participant that are distributed along the line connecting the two vowels. We then used these participant-specific speech stimuli in a standard *categorical perception task* (Möttönen and Watkins, 2009; Niziolek and Guenther, 2013). Each stimulus was presented 10 times and the order of stimuli was randomized. In each trial of the perception task, a token from the participant-specific stimuli set was presented to the participant (at 75 dB SPL) and he/she was asked to indicate (using a keypad) which word was presented (e.g., “head” or “had”; ε or æ).

### Data Analysis

The goal of this study was to examine the relationship between vowel perception and variability of vowel production. We used data from the categorical perception task and the vowel production task to drive participant-specific perception-based boundary and production-based boundary between /ε/ and /æ/.

#### Categorical Perceptual Boundary (CPB)

We fitted a logistic psychometric function to each participant’s response (proportion of /ε/ responses) using a Maximum Likelihood criterion (Kingdom and Prins, 2016; Prins and Kingdom, 2018). Evaluation of the goodness of fitted psychometric functions confirmed that the psychometric functions were fitted properly for all participants (*R^2^* > 0.85). Based on the fitted psychometric functions, we then estimated each participant’s CPB—formant values at 50% proportion /ε/-responses. We used six levels of stimuli for 27 participants and seven levels stimuli for 13 participants; our analyses did not reveal a statistically significant difference between extracted CPBs of these two groups (*p* = 0.45), and thus, we combined both groups for further analyses. Figure 1B shows the fitted psychometric functions of all participants, along with their estimated perceptual boundaries (shown as circles).

#### Variability-Based Boundary (VBB) or Center of Mass

**Figure 2:**
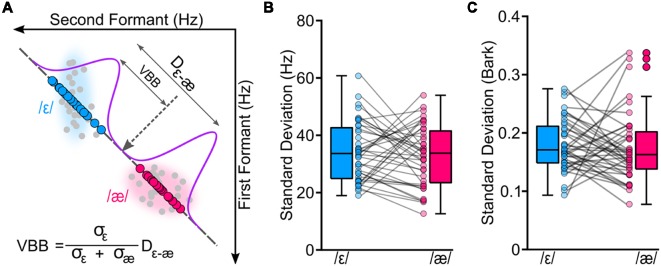
We conducted a vowel production task to estimate a theoretical variability-based boundary (VBB or the center of mass of the two vowels) between /ε/ and /æ/ for each participant. Panel **(A)** shows the original formant values (in hertz) of the two vowels for a representative participant (gray circles). To calculate VBB, we projected formant values of each participant to a line connecting the two vowels; blue circles correspond to projected formant values of /ε/, and magenta circles correspond to projected formant values of /æ/. Then, we estimated standard deviations of the vowels (σ_ε_ and σ_æ_) along with the distance between centroids of the two vowels (D_ε−æ_). Our analyses showed that two vowels had similar variabilities for formants measured in hertz (**B**; *p* = 0.507) and in bark (**C**; *p* = 0.694).

To examine variability of the two vowels, we implemented the following steps. Note that these analyses were done offline upon completion of the study, and they are different from the initial formant analysis that was done during the experimental session. First, all productions were inspected (offline) to exclude gross errors in formant tracking and to exclude trials with speech errors (e.g., producing wrong words). Approximately, 1% of all trials were excluded. Second, based on the spectrogram of each production, onset and offset of vowels were manually annotated and F1 and F2 trajectories were extracted. To extract formants, we averaged formant values from a window placed on the center of the vowel (40%–60% into the length of the vowel; steady-state portion of the vowel). Third, we projected F1 and F2 values of each produced vowel to a line connecting participant-specific median /ε/ to median /æ/. We used median /ε/ as a reference point for all projected formant values. The rationale for this procedure was to estimate the variability of the vowels along the line connecting the two vowels, as the stimuli set used in the perception task was generated along this line. Thus, this procedure ensured that vowel variability and perceptual results were along the same line and based on participant-specific vowel configurations. Fourth, given that we hypothesized that the CPB between the two vowels may co-vary with vowel variability, we used vowel variabilities to estimate a VBB. Figure 2A shows the procedure for the calculation of the VBB for one representative participant. The VBB was defined as the center of mass between the two vowel distributions, and the mass of each vowel was the inverse of its variability (standard deviation along the line connecting the two vowels). In other words, the VBB is a theoretical boundary between two vowel distributions and was calculated based on variabilities of the distributions. In these calculations, the VBB was calculated relative to the center of /ε/ (reference point).

$$VBB=\frac{\sigma_{\text{ε}}}{\sigma_{\text{ε}}+\sigma_{æ}}D_{\text{ε}-\text{æ}}$$

In this formula, σ represents the standard deviation of the vowels and *D*_ε−æ_ represents the distance between the vowel centroids. It should be noted that another approach to arrive to the same equation is based on the normalized distance of the VBB from each of the distributions. The VBB is the point between two vowels where its distance from /ε/ distribution is the same as its distance from /æ/ distribution.

$$D_{vbb-\text{ε}}=\frac{\left|VBB-\mu_{\text{ε}}\right|}{\sigma_{\text{ε}}},D_{vbb-æ}=\frac{\left|VBB-\mu_{æ}\right|}{\sigma_{æ}}$$

In these equations, μ corresponds to the mean of a vowel distribution. Given that we calculated projected formants relative to /ε/, then *μ*_ε_ = 0, *μ*_æ_ = *D*_ε−æ_, and VBB < *μ*_æ_; thus, we can simplify the equations and arrive at the equation for the center of mass.

$$D_{vbb-\text{ε}}=\frac{VBB}{\sigma_{\text{ε}}},D_{vbb-æ}=\frac{D_{\text{ε}-\text{æ}}-VBB}{\sigma_{\text{æ}}},D_{vbb-\text{æ}}=D_{vbb-\text{ε}}$$

During the study, all formants were measured in hertz and speech stimuli for the perception task were calculated in hertz. However, to ensure that the relationship between the perception and production measures were valid in psychoacoustic scales, we transformed formant values from hertz to bark (Traunmüller, 1990) and followed the same steps to estimate the VBB in bark scale. We also used a similar projection procedure to calculate the CPB in hertz and in bark for each participant. The estimated CPB and VBB values were entered in statistical analyses. Note that this study was not designed to examine whether or not perception drives production, and the association between the two systems was treated from a correlational perspective. Prior to analyses, we performed the Shapiro-Wilk test to ensure normality of all data. We used Pearson’s correlation coefficients and regression analyses to examine relationship between the VBB and the CPB. We examined residual values to confirm linear model assumptions. Additionally, we used paired *t*-tests to compare vowel variabilities. R version 3.5.1 (The R Project for Statistical Computing^1^) was used for all statistical analyses.

## Results

Table 1 shows the group average F1, F2, and projected formants in both hertz and bark. Most relevant to our analyses are the projected formant values. Note that these projected formant values were calculated relative to the center (median) of /ε/ for each participant. The average ε–æ distance (Euclidean distance in F1-F2 coordinates) in hertz was 249.09 Hz (*SD* = 80.50; 128.61–434.61), and in bark was 1.38 bark (*SD* = 0.37; 0.81–2.35). As shown in Figures 2B,C, we did not find a statistically significant difference between the variability of /ε/ and the variability of /æ/ (standard deviation of projected formants; σ_ε_ and σ_æ_) in hertz (*t*_(39)_ = 0.670, *p* = 0.507) and in bark (*t*_(39)_ = 0.396, *p* = 0.694). Estimated CPB in hertz ranged from 34.16 to 234.51 Hz (*M* = 121.62 Hz, *SD* = 52.80) and in bark ranged from 0.21 to 1.33 bark (*M* = 0.69, *SD* = 0.27). The calculated VBB in hertz ranged from 44.81 to 256.48 Hz (*M* = 128.29, *SD* = 48.73) and in bark ranged from 0.35 to 1.33 bark (*M* = 0.71 bark, *SD* = 0.25). No statistically significant difference was found between the CPB and the VBB in hertz (*t*_(39)_ = 1.945, *p* = 0.061) or in bark (*t*_(39)_ = 1.022, *p* = 0.313).

**Table 1:** Group average and standard deviation (inside parentheses) of the formant values and projected formant values for /ε/ and /æ/ in hertz and bark.

	F1		F2		Projected formants	
	Hz	Bark	Hz	Bark	Hz	Bark
/ε/	721.87 (100.66)	6.53 (0.78)	1904.99 (153.75)	12.76 (0.55)	0.15 (1.81)	0.00 (0.01)
/æ/	902.79 (131.97)	7.83 (0.91)	1731.40 (152.78)	12.13 (0.62)	249.21 (80.55)	1.39 (0.37)

*The center of /ε/ was used as the reference point for projected formant values*.

**Figure 3:**
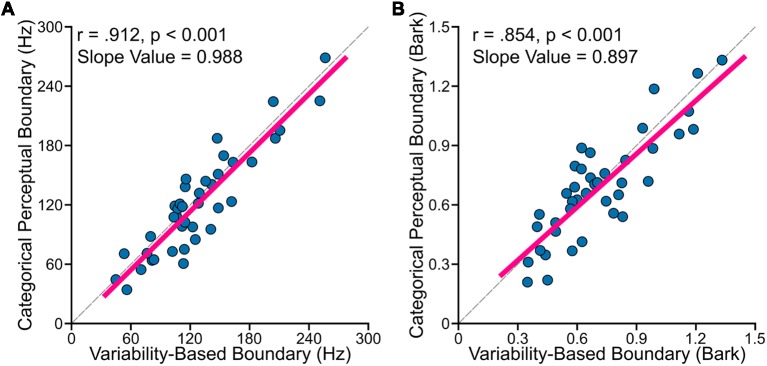
Individual participants’ data showed (**A**: formants calculated in hertz; **B**: formants calculated in bark) that VBB (or the center of mass of two vowels) strongly and positively correlated with the CPB for data measured in hertz (*r* = 0.912, *p* < 0.001) and in bark (*r* = 0.854, *p* < 0.001). The dashed lines in **(A,B)** correspond to lines with the slope of one.

The primary goal of this study was to examine relationships between perceptual boundary (measured in categorical perception task) and VBB (calculated based on data from the vowel production task). First, to test whether the CPB is farther from more variable vowels, we examined the relationship between standard deviation of /ε/ normalized by the sum of the standard deviations of /ε/ and /æ/ [i.e., σ_ε_/(σ_ε_ + σ_æ_)], using Pearson’s correlation coefficients (*r*). We found statistically significant positive correlation between the CPB and the normalized vowel variability (for data in hertz: *r* = 0.425, *p* = 0.006; for data in bark: *r* = 0.426, *p* = 0.006). This result suggested that the CPB was closer to the center of /ε/ in speakers with less variable /ε/ (relative to /æ/), and farther from the center of /ε/ in speakers with more variable /ε/. Second, as mentioned in the method section, the VBB is a theoretical boundary located between two vowels such that its distance from /ε/ distribution is the same as its distance from /æ/ distribution. In other words, the VBB is the optimal point between the two vowel distributions (i.e., the center of mass of the two distributions). Using Pearson’s correlation coefficients, we found that the CPB strongly and positively correlated with the VBB both in hertz (*r* = 0.912, *p* < 0.001) and in bark (*r* = 0.854, *p* < 0.001). As shown in Figure 3, the two methods of calculation (hertz and bark) resulted in similar outcomes. Third, we used regression analyses to: (a) estimate the slope value; and (b) examine how much of the variability of the CPB can be explained by the VBB. We conducted a simple linear regression to test whether the VBB in hertz predicted the CPB in hertz (CPB = Slope × VBB + Intercept). We found that the VBB in hertz explained 82.7% of the variance of the CPB in hertz (*R*^2^ = 0.827, *F*_(1, 38)_ = 187.3, *p* < 0.001), with a statistically significant slope value of 0.988 (*p* < 0.001). Similarly, we found that the VBB in bark explained 72.2% of the variance of the CPB in bark (*R*^2^ = 0.722, *F*_(1, 38)_ = 102.3, *p* < 0.001), with a statistically significant slope value of 0.897 (*p* < 0.001).

## Discussion

Previous studies have provided behavioral and neural evidence for the link between the speech production and speech perception systems (Galantucci et al., 2006; Tatham and Morton, 2006; Hickok, 2012; Perkell, 2012; Guenther, 2016). In the present study, we examined whether the perception of two adjacent vowels interacts with the production variabilities of the two vowels. We conducted a standard categorical perception task to estimate the CPB between /ε/ and /æ/ using participant-specific speech samples. We also conducted a vowel production task to determine participant-specific variabilities of /ε/ and /æ/. In an analogy to physics, two adjacent vowels can be considered as two connected masses, and the “mass” of each vowel can be determined by the inverse of the variability of the vowel distribution. Based on this analogy, we hypothesized variabilities of two adjacent vowels may co-vary with the categorical boundary between the vowels, and the “center of mass” of the vowel categories (VBB) correlates with the CPB. Consistent with our hypotheses, we found that: (a) the CPB was farther from more variable vowels and closer to less variable vowels; and (b) the CPB strongly correlated with the VBB, and that the VBB explained 72%–82% of the variance of the CPB.

One interpretation of these results is that the SMS uses the CPB between two adjacent vowels—in addition to auditory goals—to determine errors in its auditory output, and thus, to constrain vowel variabilities. An alternative interpretation is that our productions shape our perception, and thus, vowel production variability drives categorical perception between adjacent vowels. It should be noted that these two interpretations are not mutually exclusive. This study was not designed to examine whether or not perception drives production, and the association between the two systems was treated from a correlational perspective; therefore, our results cannot rule out any of these interpretations. Empirical results and theoretical frameworks have shown that we acquire our auditory goals during infancy and childhood, and then, we use the acquired auditory goals to drive the speech production system (Callan et al., 2000; Kuhl, 2004; Guenther and Vladusich, 2012; Guenther, 2016). However, this mechanism may be different during adulthood. Based on our findings and previous reports of the close association between the perception and production systems (Tatham and Morton, 2006; Guenther, 2016), we propose that the link between speech perception and speech production is dynamic and the two systems bi-directionally influence each other. In this view, after the speech acquisition stage, the perception system and the production system seamlessly “converge” together. Therefore, change in one system could result in change in the other system—although the required magnitude and duration of exposure to a change in one system to result in a similar change in the other system is not necessarily equal for the two systems. One outcome of the convergence of the two systems is that the categorical perceptual boundary and production variability change until they reach an equilibrium at which the categorical boundary is located at the most optimal point between the two vowels. The position of this optimal point is related to both the Euclidian distance and the variability of vowels (defined as the VBB or the center of mass in this study).

Our results are largely in agreement with previous studies that have examined the relationship between speech production and perception (Newman, 2003; Perkell et al., 2004b, 2008; Nieto-Castanon et al., 2005; Franken et al., 2017). Such studies have typically used discrimination tasks to find perceptual acuity of a given vowel. The rationale for using discrimination tasks is primarily based on theoretical frameworks of speech production (Perkell et al., 1997; Guenther and Vladusich, 2012; Perkell, 2012; Guenther, 2016). For example, the Directions Into Velocities of Articulators (DIVAs) model of speech production suggests that speech units are partially represented as auditory goals, and that the auditory feedback during speech production is compared to the auditory goals (Guenther, 2016). If there is a discrepancy between the auditory goals and the incoming auditory feedback, then the brain issues a corrective motor command to compensate for the error. Based on this account of speech production, speakers with smaller auditory goals would be more sensitive to errors, which could lead to more precise and consistent speech production (i.e., less variable speech). Thus, this interpretation implies that variability of a given vowel is solely related to auditory goals of the vowel, and characteristics of adjacent vowels may not affect the vowel variability. However, there is emerging evidence (Mitsuya et al., 2011; Niziolek and Guenther, 2013; Bourguignon et al., 2014, 2016; Lametti et al., 2014b; Reilly and Pettibone, 2017) that speakers are more sensitive to experimentally induced auditory errors (through formant perturbations) that are more similar to adjacent vowels, suggesting that the SMS may also calculate “categorical errors”—i.e., whether or not the received auditory feedback of a vowel is within the vowel’s perceptual category. If this is the case, then the CPB between vowels may also serve as a boundary (or a constraint) for vowel variability (i.e., productions can be variable as long as they are within the perceptual category of the vowel). In other words, for two adjacent vowels to remain perceptually distinct, if one vowel becomes more variable, then the adjacent vowel needs to become less variable to keep the two vowels distinct. Overall, our finding of a strong relationship between the VBB and the CPB supports the view that the SMS may also use the CPB (in addition to auditory acuity) to calculate auditory errors which in turn determines/limits variabilities of adjacent vowels. This interpretation does not imply that the interaction of the perception and production is unidirectional; in fact, as we mentioned above, perception and production could influence each other dynamically and bidirectionally throughout life.

Of course, our procedure and analyses have several important limitations that require further research. First, we generated speech stimuli based on participants’ own median productions of /ε/ and /æ/, but the stimuli were generated in the F1-F2 coordinates and higher formants were not modified (e.g., F3 and F4). This may have influenced the quality of the stimuli and added some unwanted variability in the calculation of the CPB. Second, we limited our calculation to variabilities along the line connecting the two vowels (ε-æ line) for simplicity purposes; however, different vowels have different distributions and this simplification may have influenced the relationship between production variability and the CPB. Third, our study was designed to examine only two vowels, and it is unclear if this effect can be observed in other vowels. Future studies can overcome such limitations by: (a) manipulating all formants (and not just F1 and F2) to generate more accurate speech stimuli; (b) calculating the CPB and vowel variabilities along different pathways between vowels to estimate the relationship in the entire multi-dimensional formant space; and (c) examining all vowels in the English language as well as vowels in other languages.

In sum, we conducted a categorical perception task and a vowel production task to examine whether vowel perception correlates with vowel production variability. We found that the categorical boundary was farther from more variable vowels and closer to less variable vowels. Additionally, we found that the center of mass of two vowels (a theoretical boundary calculated based on production variability) strongly and positively correlated with the categorical boundary and it explained 72%–82% of the variance of the categorical boundary. Overall, our findings support a view that the speech perception and speech production systems are strongly and bidirectionally linked.

## Data Availability

The datasets generated for this study are available on request to the corresponding author.

## Author Contributions

AD designed the experiment. S-CC, DO, and AD conducted the experiment, interpreted the results and wrote the manuscript. S-CC and AD analyzed the data.

## Conflict of Interest Statement

The authors declare that the research was conducted in the absence of any commercial or financial relationships that could be construed as a potential conflict of interest.

## Footnotes

^1^www.R-project.org
